# Flecainide overdose – support using an intra-aortic balloon pump

**DOI:** 10.1186/1471-227X-5-10

**Published:** 2005-12-12

**Authors:** Jonathan Timperley, Andrew RJ Mitchell, Peter D Brown, Nicholas EJ West

**Affiliations:** 1 From the Department of Cardiac Rhythm Management, John Radcliffe Hospital, Oxford, OX3 9DU, UK; 2 From the Department Cardiology of Gloucestershire Royal Hospital, Great Western Road, Gloucester, GL1 3NN, UK

## Abstract

**Background:**

Flecainide is an antiarrhythmic agent which is being used increasingly for the management of super-ventricular arrhythmias. Overdose with flecainide is frequently fatal with mortality reported as high as 22% due to arrhythmias, myocardial depression and conduction defects leading to electro-mechanical dissociation and asytole. Supportive measures are often required during the case and previously have included inotropes, extracorporeal membrane oxygenation and cardiopulmonary bypass.

**Case presentation:**

A 47 year old lady presented to the emergency department with a four hour history of severe central chest pain. Her ECG showed atrial fibrillation and broad QRS complexes with a sine wave appearance. She had a past history of paroxysmal atrial fibrillation and significant psychiatric history. Following thrombolysis for a presumed myocardial infarction she developed cardiogenic shock with severely impaired left ventricular function. An intra-aortic balloon pump was inserted and coronary angiography demonstrated normal coronary arteries. With inotropic support she improved over 48 hours, with both her QRS duration and left ventricular function returning to normal. Biochemical testing following her discharge demonstrated significantly elevated levels of flecainide.

**Conclusion:**

The use of an intra-aortic balloon pump is a useful supportive measure during the acute phase of flecainide overdose associated with severe myocardial depression.

## Background

Flecainide is an increasingly used class 1C antiarrhythmic drug used for the management of both supra-ventricular and ventricular arrhythmias. It causes rate-dependent slowing of the rapid sodium channel slowing phase 0 of depolarisation [1] and in high doses inhibits the slow calcium channel. Flecainide also slows conduction in all cardiac fibres, increasing conduction times in the atria, ventricles, atrio-ventricular node and His-Purkinje system and can cause myocardial depression.

## Case presentation

A 47 year old lady presented to the emergency department with a four hour history of severe central chest pain radiating to the neck. She had a significant psychiatric history with multiple episodes of self harm and had previously been hospitalised with behavioural problems. The patient had a history of multiple admissions with cardiac sounding chest pain, all with normal ECG and cardiac troponin levels. An outpatient stress nuclear perfusion scan was normal. The patient also had a history of arterial hypertension and paroxysmal atrial fibrillation and was managed on a combination of amitriptyline, losartan, amlodipine and flecainide. During this admission she repeatedly denied taking an overdose.

**Figure 1:**
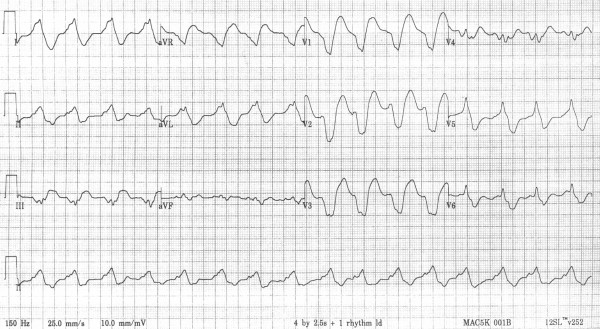
12-lead ECG on admission.

**Figure 2:**
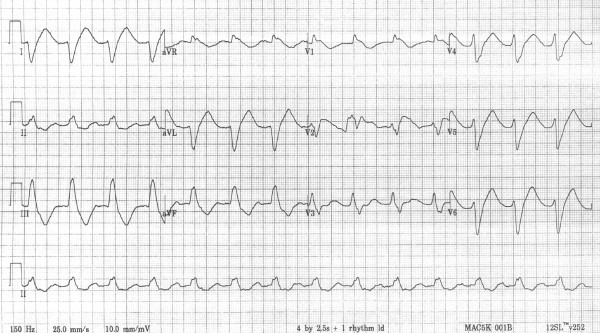
12-lead ECG after thrombolysis.

On admission to the emergency room the patient was unresponsive with a GCS of 6. She rapidly regained consciousness but remained slightly drowsy. Pupil response was normal. Her pulse was 70 bpm and blood pressure 60/40 mmHg. The 12-lead ECG (figure 1) revealed atrial fibrillation with a broad QRS complex (320 ms) with a sine wave appearance. An ECG had been performed 4 weeks previously and was normal. She had normal serum potassium (4.1 mmol/l). The presentation with chest pain led to a presumptive diagnosis of an acute myocardial infarction and she was treated with thrombolysis with intravenous alteplase. Ten minutes later there was a significant change in her ECG (figure 2). Inotropic support was instituted with dobutamine and subsequently with adrenaline to maintain her blood pressure. She remained hypotensive with poor urine output and was transferred to the regional cardiothoracic unit for further management. Blood was taken for paracetamol, salicylate levels which were normal. Flecanide levels were requested. Blood gases showed pH 7.37, pCO2 6.1kPa, pO_2 _11.7, bicarbonate 25.3 mmol/l

**Figure 3:**
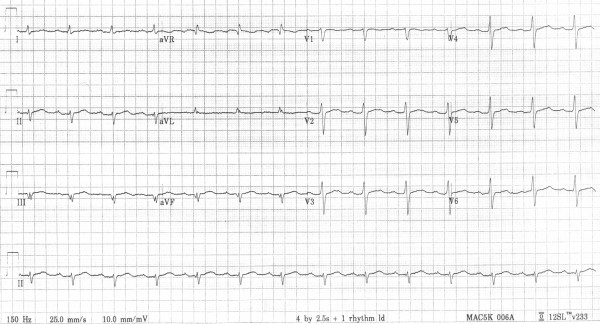
12-lead ECG after treatment.

On arrival at our institution, transthoracic echocardiography revealed global moderate impairment of left ventricular function. Emergency coronary angiography was performed demonstrating normal epicardial coronary arteries and left ventriculography confirmed the echocardiography findings. An intra-aortic balloon pump was inserted with a rapid improvement in haemodynamics. Over the following 48 hours the patient was weaned from the inotropes and the intra-aortic balloon pump was removed. During this period her QRS duration gradually returned to normal (figure 3) with left axis deviation and first degree heart block (which was present 12 months previously). Repeat echocardiography demonstrated normal left ventricular function.

Following discharge her flecanide levels were returned as 2340 μg/l (normal therapeutic range 200–700 μg/l) at 24 hours post admission.

## Discussion

The mortality of overdose with class 1C agents has been reported as high as 22% compared to less than 1% for all drug overdoses [2]. In such cases fatal outcome is often related to the progression of conductions disturbance to electromechanical dissociation and asytole. Although less common, ventricular arrhythmias may occur and are frequently unresponsive to electrical cardioversion [3]. Other features may occur due to impaired tissue perfusion including hypoxia, metabolic acidosis, coma and convulsions. Hypotension can occur within minutes of a significant flecainide overdose, and subsequent reduction in hepatic and renal flow leading to prolongation of the duration of toxicity.

The proarrhythmic effects of flecanide may be related to flecainide promoting re-entry in ventricular tissue [4]. Worsening of existing ventricular arrhythmias or the onset of new ones can occur in up to 30% of patients [5]. Flecainide depresses cardiac contractility especially in patients with underlying impairment of function.

The management of an overdose includes supportive measures and specific pharmacological agents. The benefit of activated charcoal is uncertain but this may be considered within the first hour of ingestion [8]. Hemodynamic support may be required including the fluid replacement, inotropes and an intra-aortic balloon pump as in this case. Both extracorporeal membrane oxygenation and cardiopulmonary bypass have been used in cases of severe overdose [6,7] but the use of intra-aortic balloon pump has not previously been described.

Sodium bicarbonate is recommended for a metabolic acidosis that persists despite correction of hypoxia and adequate fluid resuscitation [8]. Following administration of intravenous sodium bicarbonate QRS narrowing has been reported and also termination of ventricular tachycardia. [9]. Ventricular arrhythmias may be difficult to cardiovert electrically and both lidocaine and amiodarone have been used successfully in such cases [3,10]. Cardiac pacing may be required but ventricular capture may be poor [3].

## Conclusion

Flecainide overdose is frequently fatal and supportive measures including the use of intra-aortic balloon pumping may be required for severe myocardial depression that may occur during the acute phase.

## Competing interests

The author(s) declare that they have no competing interests.

## Authors' contributions

JT, AM and PB drafted the manuscript. NW made final alterations to manuscript.

## Pre-publication history

The pre-publication history for this paper can be accessed here:


